# Let fever do its job: The meaning of fever in the pandemic era

**DOI:** 10.1093/emph/eoaa044

**Published:** 2020-11-23

**Authors:** Sylwia Wrotek, Edmund K LeGrand, Artur Dzialuk, Joe Alcock

**Affiliations:** 1 Department of Immunology, Nicolaus Copernicus University, Torun, Poland; 2 Department of Biomedical and Diagnostic Sciences, College of Veterinary Medicine, University of Tennessee Knoxville, TN, USA; 3 Department of Genetics, Kazimierz Wielki University, Bydgoszcz, Poland; 4 Department of Emergency Medicine, University of New Mexico, Albuquerque, USA

**Keywords:** fever, antipyretic, acetaminophen, host defense, COVID-19, sickness behavior

## Abstract

Although fever is one of the main presenting symptoms of COVID-19 infection, little public attention has been given to fever as an evolved defense. Fever, the regulated increase in the body temperature, is part of the evolved systemic reaction to infection known as the acute phase response. The heat of fever augments the performance of immune cells, induces stress on pathogens and infected cells directly, and combines with other stressors to provide a nonspecific immune defense. Observational trials in humans suggest a survival benefit from fever, and randomized trials published before COVID-19 do not support fever reduction in patients with infection. Like public health measures that seem burdensome and excessive, fevers involve costly trade-offs but they can prevent infection from getting out of control. For infections with novel SARS-CoV-2, the precautionary principle applies: unless evidence suggests otherwise, we advise that fever should be allowed to run its course.

Lay summary: For COVID-19, many public health organizations have advised treating fever with medicines such as acetaminophen or ibuprofen. Even though this is a common practice, lowering body temperature has not improved survival in laboratory animals or in patients with infections. Blocking fever can be harmful because fever, along with other sickness symptoms, evolved as a defense against infection. Fever works by causing more damage to pathogens and infected cells than it does to healthy cells in the body. During pandemic COVID-19, the benefits of allowing fever to occur probably outweigh its harms, for individuals and for the public at large.

## INTRODUCTION

Although fever appears many times during our lives, its utility is often ignored. This is reflected in recent observations concerning COVID-19, caused by the SARS-CoV-2 virus. Several authors have raised and discussed the concern that certain drugs that upregulate ACE2 receptors, such as ACE inhibitors and non-steroidal anti-inflammatory drugs (NSAIDs), might make the infection worse [1–3]. Less attention has been given to the possibility that fever suppression by antipyretics itself carries risks in the setting of an acute infectious illness, such as COVID-19 [4, 5]. Fever is one of the most common presenting symptoms of COVID-19, occurring in approximately 80% of cases [6]. Major health organizations have issued inconsistent and sometimes contradictory guidance for fever treatment during COVID-19. Guidelines have variously advised taking antipyretics for fever reduction [7, 8], recommended antipyretics, e.g. acetaminophen, ‘to feel better’ without mentioning fever [9], provided reassurance that antipyretics, e.g. NSAIDS, are not harmful [7, 10, 11], and recommended antipyretics for fever and other symptoms, but not solely to reduce body temperature [12]. Against this backdrop, a large literature exists on fever and associated symptoms as a defense against infections [13–20], and previous researchers have warned against the routine use of antipyretics during infections [21–27]. Now we find that this concern must be revisited for COVID-19, an often-lethal disease for which we currently have no cure or vaccine.

In this review, we outline the role of fever during infections, and we consider whether fever is beneficial, harmful or neutral to the host and to the pathogen. We review the evidence for and against interventions that lower or raise an infected patient’s temperature. Additionally, we discuss how population-level approaches to fever might affect public health. In three text boxes, we provide more detailed context from an evolutionary medicine viewpoint.

## FEVER AND SICKNESS SYMPTOMS

Fever is part of a systemic response to infection known as the acute phase response (Box 1). Fever itself is the controlled elevation of body temperature due to the upward resetting of the hypothalamic thermostat. This distinguishes fever from hyperthermia, e.g. heat stroke, which involves overheating of the body while the temperature set-point remains normal. More rarely, especially in very severe infections, there may be a controlled depression of body temperature, termed anapyrexia [28], to be distinguished from unregulated hypothermia. Fever is perceived as alarming and uncomfortable in early stages since the individual feels cold and may shiver. And fever is uncomfortable when it breaks, since the individual feels uncomfortably warm and may sweat. As part of the acute phase response, fever is almost invariably accompanied by uncomfortable sickness symptoms and behaviors, notably lethargy, depression and aches. Although fever and sickness symptoms/behaviors are regulated in different regions of the brain, both are inhibited by antipyretic drugs (e.g. NSAIDs and acetaminophen) [29, 30]. The NSAIDs, particularly ibuprofen, are considered to have more anti-inflammatory properties than acetaminophen and may carry additional risks of promoting infection [31].

## HOW FEVER WORKS—EXPERIMENTS AND THEORY

Fever is widespread throughout the animal kingdom. In addition to the internally generated fevers of mammals and birds, many invertebrates and poikilothermic vertebrates have been observed to raise their body temperature behaviorally, although baseline and febrile temperatures vary substantially depending on the species [32, 33]. Likewise, pathogens are expected to be adapted to the temperature of their host [20], and it has been noted that most fungi are precluded from infecting mammals and birds due to high endothermic temperatures [34]. The subsequent discussion of specific temperatures is primarily directed at mammalian fever with the recognition that many of the principles could apply more widely to other species and their pathogens.

Fever’s ability to protect against infection has been well established through numerous *in vitro* and *in vivo* experiments and has been extensively reviewed [13, 14, 16, 17, 23]. A variety of infection-fighting mechanisms have been proposed for fever, though the relative importance of each mechanism remains to be established. Febrile temperatures enhance a variety of immune cells functions, reviewed in [13] and [14]. These include motility, phagocytosis and reactive oxygen species production by neutrophils and monocytes, as well as enhanced function of natural killer cells, dendritic cells, T-helper cells and antibody-producing cells. Febrile temperatures increase type I interferon responses [35], notable here because interferons have antiviral activities, and reduced type I interferon activity is associated with severe COVID-19 disease [36]. In addition, fever can induce heat shock proteins in both pathogens and host cells, resulting in downstream induction of adaptive and innate components of the host immune response [17, 37]. Fever also increases the vulnerability of rapidly dividing pathogens to destruction, acting in concert with other stresses such as iron deprivation [38] and the effects of antibiotics [39]. Very elevated temperatures (e.g. 42°C) are synergistic with reduced pH and hypoxia in killing of mammalian cells [40]. The combined actions of heat-enhanced performance of immune cells plus heat-induced stress of pathogens (including infected cells), along with other stressors, highlight fever’s multilayered defense likely being greater than the sum of its parts.

Clint and Fessler [20] pointed out another theoretical benefit to fever. They note that ‘pathogen strains that are more fever-tolerant are consistently outcompeted by strains that are better optimized for normal body temperature and are thus more successful at infecting non-febrile hosts’. They propose that fever is beneficial in defense by widening the differences between the pathogen life-history trade-offs between growth in infected hosts versus transmission to new hosts. Because fever-adapted pathogens pay a fitness cost at normal host body temperature, between-strain competition may explain the wide phylogenetic distribution of fever and its evolutionary persistence as a host strategy.

## WHY PEOPLE TAKE ANTIPYRETICS

Antipyretics are taken by some people in order to treat sickness symptoms, such as headache, malaise and muscle aches. Others take antipyretics out of concern that the high temperature itself is harmful. Patients can misunderstand the source of fever, believing that it is caused directly by the infection, instead of the body’s response to infection. Some patients and medical professionals consider fever to be dangerous and take or prescribe antipyretics to return the body temperature to normal. The excessive fear of fever has been termed fever phobia [41, 42]. Parents in particular are often anxious that their children’s high temperature will cause a seizure. Febrile seizures are frightening events that occur mostly in children younger than 6 years of age. Fortunately, most common febrile seizures are harmless and leave no neurological sequelae [43, 44]. In addition, there is no evidence that taking an antipyretic will prevent the occurrence of a febrile seizure [43].

Because of the ubiquity of prescribed and over-the-counter NSAIDs and acetaminophen, these drugs may be assumed to be benign. In many cases, blocking fever and sickness symptoms is inconsequential because most infections are self-limiting, as is typical for the common cold. For potentially lethal infections like COVID-19, interfering with evolved defenses may be more problematic.

## THE BENEFITS AND COSTS OF FEVER

Historically, fever has been used therapeutically in ways that highlight its potential benefits and costs. Before the advent of antibiotics, severe neurosyphilis was successfully treated by infecting patients with *Plasmodium vivix* malaria to induce fever [45, 46]. In similar fashion, there are reports of certain cases of cancer treated successfully with live or killed bacteria that stimulate fever, a therapy once known as Coley’s toxin [47, 48]. These therapies carry risks, and like many interventions, they can be dangerous. Evolved host defenses like fever involve costs and risks also, but those risks must be taken in context.

Somewhat counterintuitively, the harms of fever may be central to its success as a defensive strategy [49]. Analogous to the risks of chemotherapy against rapidly replicating cancer cells, the body induces harm to both self and pathogen, gambling that it can endure those costs more than the pathogens (or pathogen-containing cells). This gamble, termed ‘immune brinksmanship’, [18, 49] has typically paid off during evolution for humans and other animals, supported by the ubiquity of fever and the acute phase response across much of the animal kingdom. This viewpoint implies that treatments that reduce fever and sickness symptoms might make a patient feel better at the cost of long-evolved defense and potentially worsen outcomes during infections.

## ASSOCIATION OF FEVER WITH FAVORABLE OUTCOMES—OBSERVATIONAL STUDIES

Building on previous theoretical and basic scientific research on fever, recent observational trials in humans have examined the use of antipyretics and fever on disease outcomes. Inhibition of fever with acetaminophen has been linked with delayed recovery, including from chickenpox and malaria [50, 51]. The use of NSAIDs has been linked with complications, including empyema and prolonged hospitalization, in children and adults with lower respiratory tract infections, reviewed in [52]. Potentially beneficial effects of fever have also been reported in observational trials. In a prospective trial that examined the effect of mitochondrial genetic variants in patients with sepsis, the mitochondrial haplotype H was linked with higher febrile temperatures and improved survival; overall, the best survival occurred in those with the highest core temperature within the first 24 h [53]. In two studies of sepsis and severe infection in Sweden and Denmark, each involving more than 2000 patients, fever was associated with lower mortality, and those with the highest body temperatures had the best survival [54, 55]. Young *et al*. [56] showed that lower temperatures were associated with higher mortality among a cohort of 269,078 ICU patients with infection in New Zealand and Australia, and the same result was seen in a cohort numbering 366, 973 in the UK. In a recent observational study of patients with COVID-19 pneumonia, it was found that having a fever (≥39°C) was associated with better survival [57], although another study involving COVID-19 showed a positive association between fever and more severe cases [58].

## FEVER SUPPRESSION DOES NOT IMPROVE OUTCOMES—HUMAN INTERVENTIONAL STUDIES

Evidence from randomized controlled trials suggests that intervening to reduce fever does not improve patients’ survival. In the HEAT trial, Young *et al*. [59] randomized critically ill patients to fever suppression with acetaminophen versus a group with fever but not receiving acetaminophen. No improvement in survival was seen in the acetaminophen group, but no clear harm was seen either. In a randomized controlled trial in ICU patients, ibuprofen did not improve survival [60]. Some human trials have shown harm from reducing the body temperature lower than normal in critically ill patients with infection. In 2013, a randomized, although not blinded, trial using chilled intravenous saline to induce hypothermia in patients with meningitis was stopped early after an interim review revealed increased mortality in the intravenous cooling group [61]. A more recent trial studied mechanical cooling of critically ill patients with septic shock. This trial randomized patients to induced hypothermia using external chilled circulating water, compared to those receiving usual care, which included some medications such as acetaminophen. The trial was also terminated early after enrolling 436 of a planned 560 patients; interim analysis pointed to higher mortality in the mechanical cooling group [62]. A meta-analysis of randomized controlled trials comparing aggressive treatment of fever versus usual care found no survival benefit from aggressive fever reduction [63]. Even medically fragile patients, including those with heart disease and limited physiological reserves, did not benefit from intensive efforts to reduce body temperature [63]. Taken together, these studies suggest that survival outcomes of patients with infection are not improved by interventions used to lower body temperature and suppress fever. On the contrary, randomized trials of body temperature reduction using intravenous or mechanical cooling have shown a signal of harm. These results are in line with increased mortality seen in observational trials [56] and suggest that interfering with the physiologic set point for body temperature can have unintended consequences.

## THE EFFECT OF ANTIPYRETICS ON ANTIBODY RESPONSES TO INFECTION OR VACCINES

When given with vaccinations, antipyretics have been observed to decrease post-vaccination muscle soreness and fever. Concerns have been raised that antipyretics might simultaneously reduce the production of antibodies after vaccinations and might have a similar deleterious effect during infections. In a small study of rhinovirus-infected volunteers, aspirin and acetaminophen both suppressed antibody responses [64]. Several large vaccine trials have reported statistically significant reductions in antibody levels when antipyretics were used at the time of vaccination [65–68]. In contrast, several studies have reported no effect of antipyretics on antibody production [69–71]. When it occurs, the mechanism for inhibited antibody responses involves the enzyme cyclooxygenase-2, which is upregulated by antibody-producing B cells [72] and inhibited by NSAIDs and acetaminophen [73]. The potential for NSAIDs and acetaminophen to reduce protective antibodies will remain a concern for COVID-19 infection and for any future vaccines for this disease.

## PUBLIC HEALTH CONSIDERATIONS

We see three important public health considerations relating to inhibition of fever and sickness symptoms. First, fever is used to monitor and restrict the spread of epidemics in the population (e.g. screening for entry into buildings) and for monitoring recovery status of patients. By masking high temperatures, antipyretics reduce the effectiveness of these public health measures. Second, if people feel fine after taking antipyretics while still shedding virus, they are a threat for spreading the infection [74]. In discussing the possibility of asymptomatic transmission in COVID-19, Han and Yang [75] noted a published report of asymptomatic spread of the virus from what was thought to be the index case in Germany. In fact, the index case had taken an antipyretic for a fever, highlighting the potential for these medications to make the disease more difficult to detect. Third and most important, there is real concern about patient morbidity and mortality due to abrogation of host defenses, which remain inadequately understood. The protective value of fever may be slight but crucial for individual patients. In a pandemic involving millions, even a modest protective effect can affect large numbers of patients.

## EARLY DEFENSE TO ‘FLATTEN THE CURVE’ IN THE INFECTED INDIVIDUAL

Analogous to epidemics affecting populations, early-stage infections within individuals exhibit exponential growth of pathogens and/or pathogen-infected cells (Table 1). As we have recently learned, timing is everything when introducing control measures in a pandemic. Early interventions have a disproportionate effect on exponential growth and outcomes. Early initiation of host defenses in individuals is similarly important. At the early part of the pathogen growth curve, the benefit of control measures may not be apparent to the observer and the costs may seem excessive. Just as with an epidemic, the question arises for an individual’s immune defenses: ‘how much of a threat exists and how vigorous and costly should the response be?’ While there are costs in overreacting, an absent or insufficient immune response to infection can be far more costly to the individual [76].

**Table 1.** Similarities between epidemics and individual infections

Features in common
Exponential increase of cases (epidemics) and of pathogens (infections)
Early intervention is best
Early control efforts can seem excessive and unwarranted
Failure of early control greatly increases costs (both due to the infections/pathogens themselves and due to the control measures)
Terminating control efforts early leads to setbacks
Complete eradication of the disease can be costly

The current COVID-19 pandemic has increased awareness of basic epidemiologic principles and the concept of ‘flattening’ the exponential growth curve. By slowing the spread of infection, the increasingly and overwhelmingly rapid spread is avoided, potentially ending the epidemic or at least gaining valuable time to develop sophisticated defenses and vaccines. Likewise for flattening the curve in newly infected individuals. Defenses like fever that are deployed early have the potential to favorably alter the trajectory of infection, providing time until adaptive immune defenses (e.g. cytotoxic T lymphocytes and antibodies) can respond. Much attention has been devoted to the difficulty of treating late-stage COVID-19 patients. In these patients, early-stage immune defenses may have been inadequate to control the infection [36], resulting in later defenses that can be harmful themselves [77, 78]. With COVID-19, Shi *et al*. [79] suggest that stronger early immune efforts are key to avoiding overwhelming late-stage disease. Similarly, fever and the acute phase response may prevent late sequelae of infection.

## PERMISSIVE APPROACHES TO ABNORMAL PATIENT PARAMETERS

**Figure 1.**
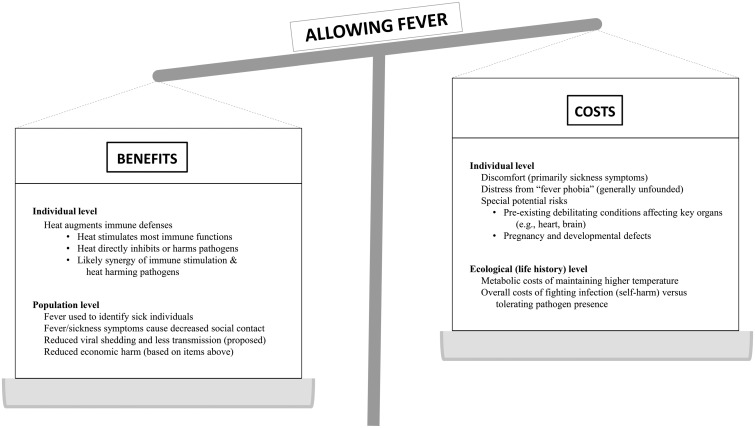
Letting fever run its course—benefits and risks

We have discussed the benefits and costs of allowing fever to occur (Fig. 1). We note that with technological advances it is possible and therapeutically tempting to ‘correct’ abnormal patient parameters all types. Several decades of intensive care experience have shown that many aggressive interventions aimed at restoring homeostasis do not improve outcomes [80, 81]. Reduced intensity of treatment has produced equivalent or better patient outcomes in a variety of clinical settings, including mechanically ventilated patients receiving lower than normal tidal volumes [82], avoiding supplemental oxygen in patients with acute myocardial infarctions [83], less aggressive nutritional supplementation in patients receiving intensive care [84], and abandoning early goal-directed therapy in sepsis [85]. The risks of enhancing infection by restoring blood iron levels to ‘normal’ when the levels have been reduced by the anemia of inflammation has long been known [86]. Similarly, aggressive measures to normalize body temperature, compared to usual care, have not improved outcomes in febrile patients [63]. Even usual care, the typical treatment of fever with NSAIDs and acetaminophen, is not evidence-based, raising questions for infection outcomes that could be answered by new research. Whether to raise temperatures therapeutically is another unanswered question. Subnormal temperatures of severely septic patients are often therapeutically raised towards ‘normal’, but often anapyrexia is self-limiting, and it is possible that transient anapyrexia may function to mitigate the downstream costs of a vigorous immune response [87].

## CONCLUSIONS

People with infections have recurring concerns and seek medical guidance for fever, and also for aches and pains, depression, fatigue, and reduced appetite. We note the curious situation that biomedical science can promptly determine the exact genotype of infectious agents through advances in molecular biology, but we are still in the dark regarding our most ancient evolved responses to infection. Since there is no currently accepted understanding of how the numerous seemingly harmful components of the acute phase response function together in defense, this should be a long-term research priority. More urgently, this pandemic is an opportunity to undertake a randomized controlled study on the effect of antipyretics on COVID-19 outcomes. Until that time, medical decision making should be guided by the precautionary principle to take reasonable steps to reduce risk to patients [88]. Practitioners should consider the likely protective role of fever, weighed against the need to treat pain and discomfort. Especially with COVID-19, in which we are almost exclusively relying on intrinsic host immune defenses to resolve the infection, we propose erring on the side of not intervening with anti-pathogen defenses like fever. In the absence of evidence definitively showing their harm in specific situations, it would be prudent in a life-threatening infection to take advantage of all of our evolved defenses.

Box 1. The acute phase response

In addition to the local inflammatory response to infection, the systemic defensive responses to infection are known as the acute phase response [13, 89]. Besides fever, other components include mobilization of leukocytes; production of a variety of protective proteins (acute phase proteins); reduced blood levels of iron, zinc, and manganese; reduced erythrocyte production (beyond simple iron deficiency); reduced appetite (anorexia); breakdown of muscle protein and fat (cachexia or hypercatabolism); and the uncomfortable, motivation-sapping sickness symptoms and behaviors we associate with infection, including lethargy, depression and aches. The acute phase response is induced and regulated by the infected individual’s own pro-inflammatory cytokines and other mediators acting on specific cell receptors. Because these responses are initiated by the host, not by the pathogen, and because they are evolutionarily conserved—appearing in all vertebrates and many invertebrates [32, 33]—the acute phase response is considered an adaptive non-specific response to infection.

While some components of the acute phase response are generally accepted to be beneficial, other acute phase responses—lassitude, anorexia and cachexia—can seem more harmful than beneficial and their function has been debated [16, 18, 90–92]. Each of the components of the acute phase response involves either self-harm or the expenditure of limited resources. This includes manufacturing acute phase proteins and supporting an increased metabolic rate. Indeed, in humans, a 2°C higher febrile temperature uses about 20% more energy than that used at normal temperature [17, 93].

The most widely-cited explanation for these elements of the acute phase response was proposed by Hart [16] and extended by Straub *et al*. [92]. This hypothesis centers around the need to conserve resources and to reallocate energy resources towards supporting an effective immune defense. Resources are conserved by restricting less essential activities and not foraging for food. Another hypothesis is that replicating pathogens can be especially vulnerable to many of the harmful components of the acute phase response, so that the harm involved is directed more to pathogens than to the host [18, 49]. In this view, reduced appetite is a nutritional strategy that disproportionately starves pathogens of energy and micronutrients. A recently proposed additional hypothesis views sickness behavior as an evolved defense that primarily benefits close relatives. In this view, termed the ‘inclusive behavioral immune system’, social withdrawal and self-isolation prevent infection from spreading to relatives who share genes with an infected individual [91, 94].

Box 2. What temperature do pathogens actually experience?

Many potential pathogens can survive and function over a wide range of temperatures cooler than their optimum. Temperatures that are slightly higher than the optimum can damage proteins (including enzyme function), membrane lipids, and RNA and disrupt DNA synthesis in the cells of both hosts and pathogens [20, 95, 96]. For cultured mammalian cells without preconditioning, a sharp increase in lethality occurs above 43°C, with mitosis and the S-phase of the cell cycle being most sensitive [96, 97].

However, one of the concerns about the efficacy of fever in harming pathogens is that febrile temperature (i.e. core body temperature) rarely exceeds 40–41°C in humans, if that. So how can the heat of fever be expected to harm or even kill most host-adapted pathogens [95]? And furthermore, why should we expect that the heat will harm the pathogens (including virally infected cells) more than the host [18]?

The temperature to which pathogens at the infected site are actually exposed is currently unknown [18, 98]. However, it is almost certainly higher than that of the blood entering the infected site since heat is generated at inflammatory foci. For example, studies assessing the temperature of inflamed atherosclerotic plaques have found temperatures up to 2°C higher than core temperature [99, 100]. It has been proposed that one source of this heat is from the macrophages in these inflamed plaques that have upregulated levels of mitochondrial uncoupling protein 2, which generates heat rather than ATP [101]. Neutrophils activated to undergo the respiratory burst (as with phagocytosis) generate substantial heat [102–104], as expected from the oxidative reactions that produce reactive oxygen species. Likewise, blood mononuclear cells activated by interleukin-2 or interferon-γ rapidly generate heat [105]. That high temperatures can be generated very locally was revealed when mitochondrial temperatures were found to be as high as 50°C as measured by temperature-sensitive mitochondrial dyes [106].

LeGrand and Day [18] proposed that since growth and replication are universally sensitive to disruption by stressors of any kind, replicating pathogens (and infected cells generating pathogens) localized at the infected site would tend to be more vulnerable to heat stress than non-replicating host cells (stromal cells or infiltrating effector immune cells). Immune cells recruited to the site of infection (e.g. neutrophils, macrophages and lymphocytes) are routinely exposed to the high temperatures at infected sites (and themselves participate in heat generation). Therefore, it is not surprising that the optimal functional temperature of activated leukocytes is higher than normal core body temperature. In this view, fever provides a crucial temperature boost to locally warmed tissues at infected sites, elevating the temperature to the level that damages pathogens. Additionally, the systemic febrile temperature may impair replication of pathogens that have spread. This is analogous to iron deprivation as a host defense during infection: systemically iron is mildly restricted, but locally at the infected site it is much more restricted, and limited even further within phagolysosomes [18].

Box 3. The trade-offs of immune brinksmanship

All immune defenses involve important costs and benefits. The term ‘immune brinksmanship’ was coined as a metaphor for the trade-off that the host faces between self-directed costs of immunity and harm directed towards pathogenic microbes and infected host cells [49]. The benefits of the acute phase response typically outweigh its costs, because fever and other nonspecific stressors exploit the vulnerabilities of rapidly dividing pathogens [49]. As a result, costs are preferentially imposed on pathogens instead of healthy host cells. Some pathogens have counteradaptations that protect against acute phase response stresses, but these impose trade-offs themselves for pathogens. Microbe-derived heat shock proteins, for instance, impair pathogen replication and trigger additional immune responses from the host [37].

LeGrand and Alcock [49] identified a number of conditions where immune brinksmanship may be a losing strategy for an infected host. One example is having insufficient metabolic, nutritional or physiologic reserves needed to survive the stress. Having comorbidities, such as heart failure or impaired lung function, also reduce the potential payoff. Other threats to the host, such as having co-infection with another pathogen or a recent previous infection, also decrease the odds of success. Other costs specific to fever include harming tissues with rapid growth, such as during spermatogenesis in the testes [107] and embryonic and fetal development [108]. Additionally, some specialized pathogens may be relatively resistant to stresses imposed by the host.

Old age is a risk factor for respiratory failure and death in COVID-19, and in some of these cases, the costs of the immune response may exceed its benefits [109]. Proximate explanations include attenuated immune responses in the aged (immunosenescence) [110] or excessive innate immune activation (inflammaging) [111]. We note that impaired infection control may impose additional immune costs, taking the patient to the brink with potentially lethal self-harm. Also, because SARS-CoV-2 is a novel virus, some lethal cases may occur because humans have had insufficient time to evolve optimal immune responses to it.

## FUNDING

This work was supported by the Polish Minister of Science and Higher Education under the program “Regional Initiative of Excellence” in 2019–2022 (grant no. 008/RID/2018/19).

**Conflict of interest:** None declared.

## References

[1] Fang L , Karakiulakis G, Roth M. Are patients with hypertension and diabetes mellitus at increased risk for COVID-19 infection? Lancet Respir Med 2020;8:e21. 32171062 10.1016/S2213-2600(20)30116-8 PMC7118626

[2] Michaud V , Deodhar M, Arwood M et al ACE2 as a therapeutic target for COVID-19; its role in infectious processes and regulation by modulators of the RAAS system. J Clin Med 2020;9:2096. 10.3390/jcm9072096 PMC7408699 32635289

[3] Datta PK , Liu F, Fischer T et al SARS-CoV-2 pandemic and research gaps: understanding SARS-CoV-2 interaction with the ACE2 receptor and implications for therapy. Theranostics 2020;10:7448–64. 32642005 10.7150/thno.48076 PMC7330865

[4] Schmitt BD , Offit PA. Could fever improve COVID-19 outcomes? Contemp Pediatrics 2020;37:8–9.

[5] Steiner AA. *Should We Let Fever Run Its Course in the Early Stages of COVID-19?* https://deepblue.lib.umich.edu/handle/2027.42/154746 (12 August 2020, date last accessed). 10.1177/0141076820951544 PMC7754817 32930066

[6] Zhu J , Ji P, Pang J et al Clinical characteristics of 3062 COVID-19 patients: a meta-analysis. J Med Virol 2020;92:1902–14. 32293716 10.1002/jmv.25884 PMC7262119

[7] World Health Organization. *Clinical Management of Severe Acute Respiratory Infection When COVID-19 is Suspected*. 2020. https://www.who.int/publications-detail/clinical-management-of-severe-acute-respiratory-infection-when-novel-coronavirus-(ncov)-infection-is-suspected (17 October 2020, date last accessed).

[8] National Health Service. *Fever in Adults*. 2020. https://www.nhsinform.scot/illnesses-and-conditions/infections-and-poisoning/fever-in-adults (17 October 2020, date last accessed).

[9] Centers for Disease Control and Prevention. *Coronavirus Disease 2019 (COVID-19). Discontinuation of Transmission-Based Precautions and Disposition of Patients with COVID-19 in Healthcare Settings (Interim Guidance)*. 2020. https://www.cdc.gov/coronavirus/2019-ncov/hcp/disposition-hospitalized-patients.html (17 October 2020, date last accessed).

[10] European Medicines Agency. *EMA Gives Advice on the Use of Non-Steroidal Anti-Inflammatories for COVID-19*. 2020. https://www.ema.europa.eu/en/news/ema-gives-advice-use-non-steroidal-anti-inflammatories-covid-19 (17 October 2020, date last accessed).

[11] Food and Drug Administration. *FDA Advises Patients on Use of Non-Steroidal anti-Inflammatory Drugs (NSAIDs) for COVID-19*. 2020. https://www.fda.gov/drugs/drug-safety-and-availability/fda-advises-patients-use-non-steroidal-anti-inflammatory-drugs-nsaids-covid-19 (17 October 2020, date last accessed).

[12] National Institute for Health and Care Excellence. *COVID-19 Rapid Guideline: Managing Symptoms (Including at the End of Life) in the Community*. 2020. https://www.nice.org.uk/guidance/ng163 (17 October 2020, date last accessed). 33497151

[13] Blatteis CM. Fever: pathological or physiological, injurious or beneficial? J Therm Biol 2003;28:1–13.

[14] Evans SS , Repasky EA, Fisher DT. Fever and the thermal regulation of immunity: the immune system feels the heat. Nat Rev Immunol 2015;15:335–49. 25976513 10.1038/nri3843 PMC4786079

[15] Harden LM , Kent S, Pittman QJ et al Fever and sickness behavior: friend or foe? Brain Behav Immun 2015;50:322–33. 26187566 10.1016/j.bbi.2015.07.012

[16] Hart BL. Biological basis of the behavior of sick animals. Neurosci Biobehav Rev 1988;12:123–37. 3050629 10.1016/s0149-7634(88)80004-6

[17] Hasday JD , Thompson C, Singh IS. Fever, immunity, and molecular adaptations. Compr Physiol 2014;4:109–48. 24692136 10.1002/cphy.c130019

[18] LeGrand EK , Day JD. Self-harm to preferentially harm the pathogens within: non-specific stressors in innate immunity. Proc R Soc B 2016;283:20160266. 10.1098/rspb.2016.0266 PMC4843660 27075254

[19] Shephard AM , Bharwani A, Durisko Z et al Reverse engineering the febrile system. Q Rev Biol 2016;91:419–57. 29562118 10.1086/689482

[20] Clint E , Fessler DMT. Insurmountable heat: the evolution and persistence of defensive hyperthermia. Q Rev Biol 2016;91:25–46. 27192778 10.1086/685302

[21] Berlim MT , Abeche AM. Evolutionary approach to medicine. South Med J 2001;94:26–32. 11213938

[22] Cunha BA. Fever myths and misconceptions: the beneficial effects of fever as a critical component of host defenses against infection. Heart Lung 2012;41:99–101; author reply 99. 22015255 10.1016/j.hrtlng.2011.07.002

[23] Kluger MJ , Kozak W, Conn CA et al The adaptive value of fever. Infect Dis Clin North Am 1996;10:1–20. 8698984 10.1016/s0891-5520(05)70282-8

[24] Knoebel EE , Narang AS, Ey JL. Fever: to treat or not to treat. Clin Pediatr (Phila) 2002;41:9–16. 11866375 10.1177/000992280204100104

[25] Purssell E. Symptoms in the host: infection and treatment model. J Clin Nurs 2005;14:555–61. 15840069 10.1111/j.1365-2702.2004.01109.x

[26] Wrotek S , Kamecki K, Kwiatkowski S et al Cancer patients report a history of fewer fevers during infections than healthy controls. J Pre Clin Clin Res 2009;3:31–5.

[27] Young PJ , Saxena MK, Beasley RW. Fever and antipyresis in infection. Med J Aust 2011;195:458–9. 22004396 10.5694/mja11.10502

[28] Mercer JB. The glossary of terms for thermal physiology. Third edition. Revised by the commission for thermal physiology of the International Union of Physiological Sciences (IUPS thermal commission). Jpn J Physiol 2001;51:245–88. 3324054

[29] Corrard F , Copin C, Wollner A et al Sickness behavior in feverish children is independent of the severity of fever. An observational, multicenter study. PLoS One 2017;12:e0171670. 28278190 10.1371/journal.pone.0171670 PMC5344311

[30] Dantzer R. Neuroimmune interactions: from the brain to the immune system and vice versa. Physiol Rev 2018;98:477–504. 29351513 10.1152/physrev.00039.2016 PMC5866360

[31] Micallef J , Soeiro T, Jonville-Béra A-P. Non-steroidal anti-inflammatory drugs, pharmacology, and COVID-19 infection. Therapie 2020;75:355–62. 32418728 10.1016/j.therap.2020.05.003 PMC7204680

[32] Gray DA , Marais M, Maloney SK. A review of the physiology of fever in birds. J Comp Physiol B Biochem Syst Environ Physiol 2013;183:297–312. 10.1007/s00360-012-0718-z 23160839

[33] Żbikowska E , Wrotek S, Cichy A et al Thermal preferences of wintering snails *Planorbarius corneus* (L.) exposed to lipopolysaccharide and Zymosan. J Invertebr Pathol 2013;112:57–61. 22985901 10.1016/j.jip.2012.08.011

[34] Robert VA , Casadevall A. Vertebrate endothermy restricts most fungi as potential pathogens. J Infect Dis 2009;200:1623–6. 19827944 10.1086/644642

[35] Lane WC , Dunn MD, Gardner CL et al The efficacy of the interferon alpha/beta response versus arboviruses is temperature dependent. mBio 2018;9:e00535. 29691338 10.1128/mBio.00535-18 PMC5915735

[36] Hadjadj J , Yatim N, Barnabei L et al Impaired type I interferon activity and inflammatory responses in severe COVID-19 patients. Science 2020;369:718–24. 32661059 10.1126/science.abc6027 PMC7402632

[37] Srivastava P. Roles of heat-shock proteins in innate and adaptive immunity. Nat Rev Immunol 2002;2:185–94. 11913069 10.1038/nri749

[38] Kluger MJ , Rothenburg BA. Fever and reduced iron: their interaction as a host defense response to bacterial infection. Science 1979;203:374–6. 760197 10.1126/science.760197

[39] Mackowiak PA , Marling-Cason M, Cohen RL. Effects of temperature on antimicrobial susceptibility of bacteria. J Infect Dis 1982;145:550–3. 7069235 10.1093/infdis/145.4.550

[40] Gerweck LE , Nygaard TG, Burlett M. Response of cells to hyperthermia under acute and chronic hypoxic conditions. Cancer Res 1979;39:966–72. 34477

[41] Bertille N , Purssell E, Corrard F et al Fever phobia 35 years later: did we fail? Acta Paediatr 2016;105:9–10. 26725574 10.1111/apa.13221

[42] Clericetti CM , Milani GP, Bianchetti MG et al Systematic review finds that fever phobia is a worldwide issue among caregivers and healthcare providers. Acta Paediatr 2019;108:1393–7. 30716166 10.1111/apa.14739

[43] Offringa M , Newton R, Cozijnsen MA et al Prophylactic drug management for febrile seizures in children. Cochrane Database Syst Rev 2017;2:CD003031. 28225210 10.1002/14651858.CD003031.pub3 PMC6464693

[44] Verity CM. Do seizures damage the brain? The epidemiological evidence. Arch Dis Child 1998;78:78–84. 9534684 10.1136/adc.78.1.78 PMC1717428

[45] Freitas DRC , Santos JB, Castro CN. Healing with malaria: a brief historical review of malariotherapy for neurosyphilis, mental disorders and other infectious diseases. Rev Soc Bras Med Trop 2014;47:260–1. 24861309 10.1590/0037-8682-0209-2013

[46] Vogel G. Malaria as lifesaving therapy. Science 2013;342:686. 24202157 10.1126/science.342.6159.686

[47] Vernon LF. William Bradley Coley, MD, and the phenomenon of spontaneous regression. Immunotargets Ther 2018;7:29–34. 29719818 10.2147/ITT.S163924 PMC5922243

[48] Wrotek S , Brycht Ł, Wrotek W et al Fever as a factor contributing to long-term survival in a patient with metastatic melanoma: a case report. Complement Ther Med 2018;38:7–10. 29857883 10.1016/j.ctim.2018.03.009

[49] LeGrand EK , Alcock J. Turning up the heat: immune brinksmanship in the acute-phase response. Q Rev Biol 2012;87:3–18. 22518930 10.1086/663946

[50] Brandts CH , Ndjavé M, Graninger W et al Effect of paracetamol on parasite clearance time in *Plasmodium falciparum* malaria. Lancet 1997;350:704–9. 9291905 10.1016/S0140-6736(97)02255-1

[51] Doran TF , De Angelis C, Baumgardner RA et al Acetaminophen: more harm than good for chickenpox? J Pediatr 1989;114:1045–8. 2656959 10.1016/s0022-3476(89)80461-5

[52] Voiriot G , Philippot Q, Elabbadi A et al Risks related to the use of non-steroidal anti-inflammatory drugs in community-acquired pneumonia in adult and pediatric patients. J Clin Med 2019;8:786. 10.3390/jcm8060786 PMC6617416 31163625

[53] Baudouin SV , Saunders D, Tiangyou W et al Mitochondrial DNA and survival after sepsis: a prospective study. Lancet 2005;366:2118–21. 16360789 10.1016/S0140-6736(05)67890-7

[54] Henriksen DP , Havshøj U, Pedersen PB et al Hospitalized acute patients with fever and severe infection have lower mortality than patients with hypo- or normothermia: a follow-up study. QJM-Mon J Assoc Phys 2016;109:473–9. 10.1093/qjmed/hcw022 26961550

[55] Sundén-Cullberg J , Rylance R, Svefors J et al Fever in the emergency department predicts survival of patients with severe sepsis and septic shock admitted to the ICU. Crit Care Med 2017;45:591–9. 28141683 10.1097/CCM.0000000000002249

[56] Young PJ , Saxena M, Beasley R et al Early peak temperature and mortality in critically ill patients with or without infection. Intensive Care Med 2012;38:437–44. 10.1007/s00134-012-2478-3 22290072

[57] Wu C , Chen X, Cai Y et al Risk factors associated with acute respiratory distress syndrome and death in patients with coronavirus disease 2019 pneumonia in Wuhan, China. JAMA Intern Med 2020;180:934–43. 32167524 10.1001/jamainternmed.2020.0994 PMC7070509

[58] Wang G , Zhang Q, Wu C et al Clinical characteristics of adult fevered COVID-19 patients and predictors for developing severe events. Front Med 2020;7:324. 10.3389/fmed.2020.00324 PMC7347790 32719804

[59] Young P , Saxena M, Bellomo R et al Acetaminophen for fever in critically ill patients with suspected infection. N Engl J Med 2015;373:2215–24. 26436473 10.1056/NEJMoa1508375

[60] Bernard GR , Wheeler AP, Russell JA et al The effects of ibuprofen on the physiology and survival of patients with sepsis. The ibuprofen in Sepsis Study Group. N Engl J Med 1997;336:912–8. 9070471 10.1056/NEJM199703273361303

[61] Mourvillier B , Tubach F, van de Beek D et al Induced hypothermia in severe bacterial meningitis: a randomized clinical trial. JAMA 2013;310:2174–83. 24105303 10.1001/jama.2013.280506

[62] Itenov TS , Johansen ME, Bestle M et al Induced hypothermia in patients with septic shock and respiratory failure (CASS): a randomised, controlled, open-label trial. Lancet Respir Med 2018;6:183–92. 29325753 10.1016/S2213-2600(18)30004-3 PMC10928558

[63] Young PJ , Bellomo R, Bernard GR et al Fever control in critically ill adults. An individual patient data meta-analysis of randomised controlled trials. Intensive Care Med 2019;45:468–76. 30741326 10.1007/s00134-019-05553-w

[64] Graham NM , Burrell CJ, Douglas RM et al Adverse effects of aspirin, acetaminophen, and ibuprofen on immune function, viral shedding, and clinical status in rhinovirus-infected volunteers. J Infect Dis 1990;162:1277–82. 2172402 10.1093/infdis/162.6.1277

[65] Doedée AMCM , Boland GJ, Pennings JLA et al Effects of prophylactic and therapeutic paracetamol treatment during vaccination on hepatitis B antibody levels in adults: two open-label, randomized controlled trials. PLoS One 2014;9:e98175. 24897504 10.1371/journal.pone.0098175 PMC4045752

[66] Li-Kim-Moy J , Wood N, Jones C et al Impact of fever and antipyretic use on influenza vaccine immune reponses in children. Pediatr Infect Dis J 2018;37:971–5. 29465480 10.1097/INF.0000000000001949

[67] Prymula R , Siegrist C-A, Chlibek R et al Effect of prophylactic paracetamol administration at time of vaccination on febrile reactions and antibody responses in children: two open-label, randomised controlled trials. Lancet 2009;374:1339–50. 19837254 10.1016/S0140-6736(09)61208-3

[68] Wysocki J , Center KJ, Brzostek J et al A randomized study of fever prophylaxis and the immunogenicity of routine pediatric vaccinations. Vaccine 2017;35:1926–35. 28262330 10.1016/j.vaccine.2017.02.035

[69] Prymula R , Esposito S, Zuccotti GV et al A phase 2 randomized controlled trial of a multicomponent meningococcal serogroup B vaccine (I). Hum Vaccin Immunother 2014;10:1993–2004. 25424809 10.4161/hv.28666 PMC4186040

[70] Sil A , Ravi MD, Patnaik BN et al Effect of prophylactic or therapeutic administration of paracetamol on immune response to DTwP-HepB-Hib combination vaccine in Indian infants. Vaccine 2017;35:2999–3006. 28449972 10.1016/j.vaccine.2017.03.009

[71] Walter EB , Hornik CP, Grohskopf L et al The effect of antipyretics on immune response and fever following receipt of inactivated influenza vaccine in young children. Vaccine 2017;35:6664–71. 29056422 10.1016/j.vaccine.2017.10.020 PMC6050004

[72] Ryan EP , Pollack SJ, Murant TI et al Activated human B lymphocytes express cyclooxygenase-2 and cyclooxygenase inhibitors attenuate antibody production. J Immunol 2005;174:2619–26. 15728468 10.4049/jimmunol.174.5.2619

[73] Bancos S , Bernard MP, Topham DJ et al Ibuprofen and other widely used non-steroidal anti-inflammatory drugs inhibit antibody production in human cells. Cell Immunol 2009;258:18–28. 19345936 10.1016/j.cellimm.2009.03.007 PMC2693360

[74] Earn DJD , Andrews PW, Bolker BM. Population-level effects of suppressing fever. Proc R Soc B 2014;281:20132570. 10.1098/rspb.2013.2570 PMC3906934 24452021

[75] Han Y , Yang H. The transmission and diagnosis of 2019 novel coronavirus infection disease (COVID-19): A Chinese perspective. J Med Virol 2020;92:639–44. 32141619 10.1002/jmv.25749 PMC7228390

[76] Nesse RM. The smoke detector principle. Natural selection and the regulation of defensive responses. Ann N Y Acad Sci 2001;935:75–85. 11411177

[77] Broggi A , Ghosh S, Sposito B et al Type III interferons disrupt the lung epithelial barrier upon viral recognition. Science 2020;369:706–12. 32527925 10.1126/science.abc3545 PMC7292499

[78] Major J , Crotta S, Llorian M et al Type I and III interferons disrupt lung epithelial repair during recovery from viral infection. Science 2020;369:712–7. 32527928 10.1126/science.abc2061 PMC7292500

[79] Shi Y , Wang Y, Shao C et al COVID-19 infection: the perspectives on immune responses. Cell Death Differ 2020;27:1451–4. 32205856 10.1038/s41418-020-0530-3 PMC7091918

[80] Kox M , Pickkers P. “Less is more” in critically ill patients: not too intensive. JAMA Intern Med 2013;173:1369. 23752755 10.1001/jamainternmed.2013.6702

[81] Vincent J-L , Singer M, Marini JJ et al Thirty years of critical care medicine. Crit Care 2010;14:311. 20550727 10.1186/cc8979 PMC2911692

[82] Determann RM , Royakkers A, Wolthuis EK et al Ventilation with lower tidal volumes as compared with conventional tidal volumes for patients without acute lung injury: a preventive randomized controlled trial. Crit Care 2010;14:R1. 20055989 10.1186/cc8230 PMC2875503

[83] Stub D , Smith K, Bernard S et al Air versus oxygen in ST-segment-elevation myocardial infarction. Circulation 2015;131:2143–50. 26002889 10.1161/CIRCULATIONAHA.114.014494

[84] Arabi YM , Reintam Blaser A, Preiser J-C. Less is more in nutrition: critically ill patients are starving but not hungry. Intensive Care Med 2019;45:1629–31. 31531714 10.1007/s00134-019-05765-0

[85] Rowan KM , Angus DC, Bailey M, PRISM Investigators et al Early, goal-directed therapy for septic shock - A patient-level meta-analysis. N Engl J Med 2017;376:2223–34. 28320242 10.1056/NEJMoa1701380

[86] Weinberg ED. Iron and susceptibility to infectious disease. Science 1974;184:952–6. 4596821 10.1126/science.184.4140.952

[87] Fonseca MT , Rodrigues AC, Cezar LC et al Spontaneous hypothermia in human sepsis is a transient, self-limiting, and nonterminal response. J Appl Physiol 2016;120:1394–401. 26989218 10.1152/japplphysiol.00004.2016

[88] Goldstein BD. The precautionary principle also applies to public health actions. Am J Public Health 2001;91:1358–61. 11527755 10.2105/ajph.91.9.1358 PMC1446778

[89] Gabay C , Kushner I. Acute-phase proteins and other systemic responses to inflammation. N Engl J Med 1999;340:448–54. 9971870 10.1056/NEJM199902113400607

[90] Schrock JM , Snodgrass JJ, Sugiyama LS. Lassitude: the emotion of being sick. Evol Hum Behav 2020;41:44–57.

[91] Shakhar K. The Inclusive Behavioral immune system. Front Psychol 2019;10:1004. 31130904 10.3389/fpsyg.2019.01004 PMC6509541

[92] Straub RH , Cutolo M, Buttgereit F et al Energy regulation and neuroendocrine-immune control in chronic inflammatory diseases. J Intern Med 2010;267:543–60. 20210843 10.1111/j.1365-2796.2010.02218.x

[93] Manthous CA , Hall JB, Olson D et al Effect of cooling on oxygen consumption in febrile critically ill patients. Am J Respir Crit Care Med 1995;151:10–4. 7812538 10.1164/ajrccm.151.1.7812538

[94] Shakhar K , Shakhar G. Why do we feel sick when infected–can altruism play a role? PLoS Biol 2015;13:e1002276. 26474156 10.1371/journal.pbio.1002276 PMC4608734

[95] González Plaza JJ , Hulak N, Zhumadilov Z et al Fever as an important resource for infectious diseases research. Intractable Rare Dis Res 2016;5:97–102. 27195192 10.5582/irdr.2016.01009 PMC4869589

[96] Hildebrandt B , Wust P, Ahlers O et al The cellular and molecular basis of hyperthermia. Crit Rev Oncol Hematol 2002;43:33–56. 12098606 10.1016/s1040-8428(01)00179-2

[97] Dewey WC , Hopwood LE, Sapareto SA et al Cellular responses to combinations of hyperthermia and radiation. Radiology 1977;123:463–74. 322205 10.1148/123.2.463

[98] Stankov S. Definition of inflammation, causes of inflammation and possible anti-inflammatory strategies. Open Inflamm J 2012;5:1–9.

[99] Casscells W , Hathorn B, David M et al Thermal detection of cellular infiltrates in living atherosclerotic plaques: possible implications for plaque rupture and thrombosis. Lancet 1996;347:1447–51. 8676628 10.1016/s0140-6736(96)91684-0

[100] Madjid M , Willerson JT, Casscells SW. Intracoronary thermography for detection of high-risk vulnerable plaques. J Am Coll Cardiol 2006;47:C80–85. 16631514 10.1016/j.jacc.2005.11.050

[101] Van De Parre TJL , Martinet W, Verheye S et al Mitochondrial uncoupling protein 2 mediates temperature heterogeneity in atherosclerotic plaques. Cardiovasc Res 2007;77:425–31. 18006489 10.1093/cvr/cvm003

[102] Eftimiadi C , Rialdi G. Increased energy expenditure by granulocytes during phagocytosis of *Staphylococcus aureus* compared with other staphylococci. J Infect Dis 1984;150:366–71. 6481184 10.1093/infdis/150.3.366

[103] Ljunggren L , Monti M, Rialdi G. A comparison of the calorimetric analysis of granulocyte activation by flow and batch systems. Thermochimica Acta 1992;207:23–8.

[104] Tan AM , Huang YQ, Qu SS. Determination of the respiratory burst of polymorphonuclear leukocytes by microcalorimetry. J Biochem Biophys Methods 1998;37:91–4. 9825302 10.1016/s0165-022x(98)00015-3

[105] Nogueira-Machado JA , Mares-Guia ML, Lima e Silva FC et al Calorimetry: a highly sensitive technique for evaluating the effect of IL-2, IFN-γ and IL-10 on the response of peripheral blood mononuclear cells. Thermochimica Acta 1999;327:57–62.

[106] Chrétien D , Bénit P, Ha H-H et al Mitochondria are physiologically maintained at close to 50°C. PLoS Biol 2018;16:e2003992. 29370167 10.1371/journal.pbio.2003992 PMC5784887

[107] Carlsen E , Andersson A-M, Petersen JH et al History of febrile illness and variation in semen quality. Hum Reprod 2003;18:2089–92. 14507826 10.1093/humrep/deg412

[108] Edwards MJ. Review: hyperthermia and fever during pregnancy. Birth Defects Res Part A Clin Mol Teratol 2006;76:507–16. 10.1002/bdra.20277 16933304

[109] Group RC , Horby P, Lim WS et al Dexamethasone in hospitalized patients with Covid-19 - preliminary report. N Engl J Med 2020:NEJMoa2021436.

[110] Gomez CR , Boehmer ED, Kovacs EJ. The aging innate immune system. Curr Opin Immunol 2005;17:457–62. 16084711 10.1016/j.coi.2005.07.013

[111] Cunha LL , Perazzio SF, Azzi J et al Remodeling of the immune response with aging: immunosenescence and its potential impact on COVID-19 immune response. Front Immunol 2020;11:1748. 32849623 10.3389/fimmu.2020.01748 PMC7427491

